# Baseline clinical characteristics and prognostic factors in hospitalized COVID-19 patients aged ≤ 65 years: A retrospective observational study

**DOI:** 10.1371/journal.pone.0248829

**Published:** 2021-03-24

**Authors:** Marta Betti, Marinella Bertolotti, Daniela Ferrante, Annalisa Roveta, Carolina Pelazza, Fabio Giacchero, Serena Penpa, Costanza Massarino, Tatiana Bolgeo, Antonella Cassinari, Marco Mussa, Guido Chichino, Antonio Maconi

**Affiliations:** 1 Infrastruttura Ricerca Formazione Innovazione, Dipartimento Attività Integrate Ricerca Innovazione, Azienda Ospedaliera “SS Antonio e Biagio e Cesare Arrigo”, Alessandria, Italy; 2 Unit of Medical Statistics, Department of Translational Medicine, University of “Piemonte Orientale” and Cancer Epidemiology, CPO Piemonte, Novara, Italy; 3 Infectious Diseases Unit, Azienda Ospedaliera “SS Antonio e Biagio e Cesare Arrigo”, Alessandria, Italy; University Magna Graecia of Catanzaro, ITALY

## Abstract

**Background:**

Individual differences in susceptibility to SARS-CoV-2 infection, symptomatology and clinical manifestation of COVID-19 have thus far been observed but little is known about the prognostic factors of young patients.

**Methods:**

A retrospective observational study was conducted on 171 patients aged ≤ 65 years hospitalized in Alessandria’s Hospital from 1^st^ March to 30^th^ April 2020 with laboratory confirmed COVID-19. Epidemiological data, symptoms at onset, clinical manifestations, Charlson Comorbidity Index, laboratory parameters, radiological findings and complications were considered. Patients were divided into two groups on the basis of COVID-19 severity. Multivariable logistic regression analysis was used to establish factors associated with the development of a moderate or severe disease.

**Findings:**

A total of 171 patients (89 with mild/moderate disease, 82 with severe/critical disease), of which 61% males and a mean age (± SD) of 53.6 (± 9.7) were included. The multivariable logistic model identified age (50–65 vs 18–49; OR = 3.23 CI95% 1.42–7.37), platelet count (per 100 units of increase OR = 0.61 CI95% 0.42–0.89), c-reactive protein (CPR) (per unit of increase OR = 1.12 CI95% 1.06–1.20) as risk factors for severe or critical disease. The multivariable logistic model showed a good discriminating capacity with a C-index value of 0.76.

**Interpretation:**

Patients aged ≥ 50 years with low platelet count and high CRP are more likely to develop severe or critical illness. These findings might contribute to improved clinical management.

## Introduction

In December 2019, a novel coronavirus, severe acute respiratory syndrome coronavirus 2 (SARS-CoV-2; previously knowns as 2019-nCoV) caused an outbreak of a febrile respiratory illness in Wuhan city, Hubei Province, China [1]. On February 11^st^, 2020 the World Health Organization (WHO) named this disease as new coronavirus disease 2019 (COVID-19) and on March 11^st^, 2020 WHO declared it a pandemic [2]. Globally, there were more than 53 million confirmed cases with a mortality rate of 3% reported to the WHO [3].

The first case diagnosed with COVID-19 in Italy (at the Sacco Hospital in Milan) was confirmed by Istituto Superiore di Sanità (ISS) on February 21^st^, 2020. Up through November 12^th^, 2020 the total number of cases has risen by more than 1 million in Italy [4].

The clinical presentation of COVID-19 is that of a respiratory illness ranging from a mild common flu-like disease to a severe pneumonia. One of the largest cohort of patients with COVID-19 showed that most cases (81%) had mild to moderate illness, 14% had severe and 5% critical illness [5]. Patients with severe COVID-19 were likely to have comorbidities such as immunosuppression, diabetes and malignancy [6]. Elderly are more susceptible to SARS-CoV-2 and 70% of elderly COVID-19 patients are more likely to progress to severe or critical illness [7]. In patients aged ≥ 80 years low oxygen saturation at admission, high C-reactive protein and elevated lactate dehydrogenase are associated with rapid progression to death [8]. Recent data show that nearly 70% of people in the U.S. were younger than 60 years and the median age of U.S. COVID-19 patients was 48 years [9]. Although younger adults are less likely than older adults to develop severe infections, 5% of all severe cases aged ≤ 50 years develop serious symptoms and complications including severe pneumonia and, more rarely, encephalitis, cardiovascular disease [10].

So far, limited data are available for younger COVID-19 patients [11] thus, this study aims to describe the baseline clinical characteristics and highlight the prognostic factors of COVID-19 patients aged ≤ 65 years admitted to “SS Antonio e Biagio e Cesare Arrigo” Alessandria’s Hospital in the Piedmont Region in northern Italy.

## Methods

### Study design and participants

This case series is part of COVID-19 Registry study, a single-center ambispective observational study carried out at “SS Antonio e Biagio e Cesare Arrigo” Alessandria’s Hospital in the Piedmont Region in northern Italy. The study was approved by the Institutional Review Board (IRB) (protocol number ASO.IRFI.20.03) with a waiver informed consent for the retrospective part. The study was conducted in accordance with the Declaration of Helsinki and the Good Clinical Practices guidelines for observational studies. All consecutive adult patients aged between 18–65 years admitted to the Alessandria’s Hospital with laboratory-confirmed COVID-19 from March 1^st^ to April 30^th^ were enrolled. Patients discharged from Emergency Department were excluded.

### Procedures

In order to detect virus infection, SARS-CoV-2 nucleic acid was identified by nasopharyngeal swab specimen in all patients by real-time reverse-transcriptase polymerase chain reaction (RT-PCR), according to WHO laboratory guidance [12].

Data of hospitalized patients was extracted from electronic medical records system (TrackCare) and paper based medical records and entered by clinical study coordinators in a dedicated electronic case report form (eCRF), specifically developed using the freely available Research Electronic Data Capture (*REDCap*) platform [13]. All data was pseudonymed according to clinical study and data protection regulations. The collected variables were the following: demographical characteristics (age, sex, residence), symptoms at onset (fever, chills, cough, conjunctivitis, rhinorrhea, headache, muscle pain, fatigue, nausea, vomiting, diarrhea, dyspnea, hemoptysis, hematemesis, ageusia, anosmia), clinical manifestations (temperature, blood pressure, oxygen saturation, heart rate, respiratory rate), comorbidities (acute myocardial infarction, chronic heart failure, peripheral vascular disease, dementia, Chronic Obstructive Pulmonary Disease (COPD), cerebrovascular disease, connective tissue disease, peptic ulcer disease, mild liver disease, diabetes, hemiplegia, moderate-to-severe kidney disease, diabetes with chronic complications, cancer without metastasis, leukemia, lymphoma, moderate or severe liver disease, metastasis, Acquired Immune-Deficiency Syndrome (AIDS)), laboratory parameters (hematology, biochemistry, blood coagulation, inflammatory markers), radiologic findings based on chest X-ray or CT scan (normal, monolateral or bilateral ground-glass opacity, interstitial involvement, irregular shading), complications (septic shock, acute respiratory distress syndrome, acute kidney injury, hemorrhages, rhabdomyolysis, pneumonia, heart failure, respiratory decompensation, hypoproteinemia, acidosis, sepsis, acute heart damage, pulmonary embolism, deep vein thrombosis, respiratory failure), treatment typologies (antibiotics, antivirals, antifungals, corticosteroids, oxygen therapy, non-invasive mechanical ventilation, invasive mechanical ventilation, ExtraCorporeal Membrane Oxygenation (ECMO), immunoglobulins, renal replacement therapy, chloroquine/hydroxychloroquine, antithrombotic prophylaxis, deep vein thrombosis/pulmonary embolism therapy), outcome (hospital discharge, transfer, death). For each patient, we calculated the Charlson Comorbidity Index (CCI) based on the available data [14]. Patient’s medical records were accessed until July 2020.

### Outcomes

According to WHO interim guidance [15], patients were divided into two groups: patients with mild or moderate disease and patients with severe or critical disease (Table 1).

**Table 1:** Classification of COVID-19 disease severity.

Group 1		Group 2	
*Mild disease*	*Moderate disease*	*Severe disease*	*Critical disease*
• Symptoms as fever, cough, fatigue, dyspnea, myalgia, headache, diarrhea, nausea/vomiting, loss of smell/taste • No pneumonia	• X-ray findings of pneumonia Blood oxygen saturation levels (SpO_2_) ≥ 90% on room air • No complications related to severe conditions	Mild or moderate clinical features plus at least one of the following manifestations:	Severe manifestations plus any other features that suggest disease rapid progression:
		• respiratory rate > 30; • breaths/minute or severe respiratory distress; SpO_2_ < 90% on room air	• respiratory failure with need mechanical ventilation; • presence of acute respiratory distress syndrome (ARDS), sepsis, or septic shock, other complications include acute pulmonary embolism, acute coronary syndrome, acute stroke, and delirium

### Statistical analysis

A forward multivariable logistic regression analysis was applied to the data in order to develop a predictive model for severe disease. Variables identified from the univariable analysis as potential predictors have been included in the multivariable analysis. A two-sided *P* value < 0.05 was considered statistically significant.

To validate the variables selection for the prognostic model, a bootstrap analysis was computed. Bootstrap-corrected Harrell’s C-index was used to assess the model’s discriminatory ability. The C-index was estimated by bootstrapping with 1000 resamples to estimate an unbiased measure of the ability of our predictive model to discriminate among patients with respect to their severe/not severe disease. Calibration was tested using the Hosmer-Lemeshow test. All analyses were performed using the programme *SAS* (Statistical Analysis Software *9*.*4*, *SAS* Institute Inc, Cary, North Carolina, USA) and STATA v.16 (*Stata* Corp, Texas, USA).

## Results

Between March 1^st^ to April 30^th^, 2020, 501 COVID-19 patients with laboratory-confirmed SARS-CoV-2 infection were hospitalized in the “SS Antonio e Cesare Arrigo” Hospital in Alessandria, Piedmont region (Italy), of whom 171 (34%) aged ≤ 65 years. The majority of young patients were males (61%) with a mean (± SD) age of 53.6 (± 9.7). On the basis of clinical manifestations 171 patients were divided into two groups: 89 cases with mild or moderate disease (group 1) and 82 cases with severe or critical disease (group 2) (Tables 2 and 3). Patients were mainly male both in group 1 (56.2%) and in group 2 (65.9%). The mean (± SD) age was higher in the group 2 (56.72 ± 6.75) than group 1 (50.85 ± 11.2). In both groups, the majority of patients aged ≥ 50 years (group 1, 60.7%; group 2, 85.4%) and presented more than one symptoms at onset (group 1, 73%; group 2, 78.1%). Charlson Comorbidity Index (CCI) showed a higher score in patients with severe or critical disease than in patients with mild or moderate disease (group 2, 1.98; group 1, 1.79). Patients aged ≥ 50 years are more likely to develop severe or critical illness (85.4% versus 60.7%, *p*<0.0001). There were no significant differences in the distribution of sex, symptoms at onset, and Charlson Comorbidity Index between the two groups.

**Table 2:** Epidemiological and baseline clinical features of hospitalized COVID-19 patients aged ≤ 65 years divided into two groups according to clinical manifestations (Group 1 –mild and moderate disease; Group 2 –severe and critical disease): Univariable logistic regression analysis.

Variable	Mild/moderate disease^§^ (n = 89)	Severe/critical disease° (n = 82)	p-value
**Epidemiological data**			
Female, n° (%)	39 (43.8%)	28 (34.1%)	
Male, n° (%)	50 (56.2%)	54 (65.9%)	0.196
Age (years), mean ± SD	50.85 ± 11.2	56.72 ± 6.75	
Age <50 years	35 (39.3%)	12 (14.6%)	
Age ≥50 years	54 (60.7%)	70 (85.4%)	**<0.0001**
**Symptoms at onset, n° (%)**			
n° ≤1	24 (27%)	18 (21.9%)	
n° >1	65 (73%)	64 (78.1%)	0.447
**Comorbidities**			
Charlson Comorbidity Index^^^, mean ± SD	1.79 ± 2.3	1.98 ± 1.61	0.536
Acute myocardial infarction, n° (%)	4 (4.5%)	3 (3.6%)	
Chronic heart failure, n° (%)	4 (4.5%)	0	
Peripheral vascular disease, n° (%)	4 (4.5%)	1 (1.2%)	
Dementia, n° (%)	0	4 (4.9%)	
Chronic obstructive pulmonary disease (COPD), n° (%)	3 (3.4%)	5 (6.1%)	
Cerebrovascular disease, n° (%)	2 (2.2%)	3 (3.6%)	
Connective tissue disease, n° (%)	1 (1.1%)	2 (2.4%)	
Peptic ulcer disease, n° (%)	1 (1.1%)	1 (1.2%)	
Mild liver disease, n° (%)	3 (3.4%)	0	
Diabetes, n° (%)	6 (6.7%)	4 (4.9%)	
Hemiplegia, n° (%)	4 (4.5%)	0	
Moderate-to-severe kidney disease, n° (%)	4 (4.5%)	5 (6.1%)	
Diabetes with chronic complications, n° (%)	4 (4.5%)	2 (2.4%)	
Cancer without metastasis, n° (%)	3 (3.4%)	6 (7.3%)	
Leukemia, n° (%)	1 (1.1%)	1 (1.2%)	
Lymphoma, n° (%)	2 (2.2%)	0	
Metastasis, n° (%)	5 (5.6%)	1 (1.2%)	

^**§,°**^at admission to the Hospital. For Charlson Comorbidity Index data were available for n = 78 patients in both groups.

^**^**^The score on the Charlson Comorbidity Index is calculated on the basis of the presence of 19 conditions, each of which is assigned a weighted score of 1, 2, 3, or 6. Higher scores indicate more coexisting conditions and a higher risk of death.

A two-sided *p value* < 0.05 was considered statistically significant.

**Table 3:** Laboratory parameters at admission of hospitalized COVID-19 patients aged ≤ 65 years divided into two groups according to clinical manifestations (Group 1 –mild and moderate disease; Group 2 –severe and critical disease): Univariable logistic regression analysis.

Laboratory findings Median (IQR)	Mild/moderate disease^§^ (n = 89)	Severe/critical disease° (n = 82)	
Leukocytes count (10^3^/mcL)	6.09 (4.46–8.14)	6.77 (4.82–9.57)	0.436
Neutrophils count (10^3^/mcL)	4.42 (2.89–5.94)	5.55 (3.65–7.59)	0.150
Lymphocytes count (10^3^/mcL)	1.03 (0.76–1.46)	0.86 (0.66–1.12)	**0.001**
Erythrocytes count (10^3^/mcL)	4.69 (4.25–5.05)	4.87 (4.41–5.24)	0.333
Hemoglobin (g/dl)	13.5 (12.15–14.8)	14.1 (12.4–15.3)	0.349
Platelets (10^3^/mcL)	206 (156–269)	196 (149–243)	0.255
Hematocrit (%)	40.6 (37.2–44.5)	42.65 (38–45.3)	0.470
Eosinophils count (10^3^/mcL)	0.02 (0.01–0.05)	0.01 (0–0.03)	0.417
Prothrombin time (PT) (seconds)	13.9 (13.05–15.1)	13.9 (13.2–14.8)	0.505
Activated partial thromboplastin time (PTT) (seconds)	30.7 (29.1–32.7)	30.6 (28.7–33.1)	0.961
Ratio	1.04 (0.99–1.11)	1.04 (0.97–1.14)	0.976
D-dimer (mcg/mL)	0.55 (0.45–0.88)	1.23 (0.76–1.9)	**0.021**
Prothrombin time (International Normalised Ratio, INR) (ratio)	1.03 (0.96–1.12)	1.03 (0.98–1.1)	0.487
Sodium level (mEq/L)	138 (136–140)	138 (136–139)	0.930
Potassium level (mEq/L)	4.2 (3.9–4.6)	4.15 (3.8–4.4)	0.240
Chlorine level (mEq/L)	102 (98–104)	102 (97–104)	0.424
Calcium level (mg/dL)	8.8 (8.5–9.1)	8.4 (8–8.8)	**0.006**
LDH (U/L)	503 (417–683)	734.5 (597–998)	**<0.0001**
AST (U/L)	25.5 (18–41.5)	42 (30–56)	0.350
ALT (U/L)	25 (17–41)	31 (22–50)	0.068
Total bilirubin (mg/dL)	0.56 (0.42–0.75)	0.52 (0.39–0.74)	0.420
C-reactive protein (mg/dL)	3.58 (1.31–8.28)	10.13 (4.07–16.53)	**<0.0001**
Ferritin (ng/mL)	352.3 (154.6–783.7)	1020 (557.3–1486)	**<0.0001**
Creatinine (mg/dL)	0.76 (0.6–0.87)	0.88 (0.72–1.03)	0.105
Troponin (ng/L)	6 (2–11)	10 (4–29)	0.337
Urea (mg/dL)	30 (24–39)	39 (30–60)	**0.013**
Direct Bilirubin (mg/dL)	0.2 (0.14–0.29)	0.2 (0.17–0.35)	0.563
Gamma glutamyl transferase (GGT) (U/L)	35.5 (21–80)	58.5 (34.5–110)	0.139
Fibrinogen (mg/dL)	536 (413–673)	625 (557.5–726.5)	**0.044**
Prothrombin (%)	93 (77.5.-102.5)	92 (85–103)	0.568

^**§**^For each variable data were available for (n =): Leukocytes (n = 88), Neutrophils (n = 88), Lymphocytes (n = 88), Erythrocytes (n = 88), Hemoglobin (n = 88), Platelets (n = 87), Hematocrit (n = 87), Eosinophils (n = 88), Prothrombin time (n = 68), Activated partial thromboplastin time (n = 55), Ratio (n = 52), D-dimer (n = 49), INR (n = 68), Sodium (n = 86), Potassium (n = 83), Chlorine (n = 43), Calcium (n = 35), LDH (n = 73), AST (n = 52), ALT (n = 81), Total bilirubin (n = 61), C-reactive protein (n = 86), Ferritin (n = 71), Creatinine (n = 83), Troponin (n = 41), Urea (n = 35), Direct Bilirubin (n = 56), Gamma glutamyl transferase (n = 36), Fibrinogen (n = 25), Prothrombin (n = 36).

°For each variable data were available for (n =): Leukocytes (n = 82), Neutrophils (n = 82), Lymphocytes (n = 82), Erythrocytes (n = 82), Hemoglobin (n = 82), Platelets (n = 82), Hematocrit (n = 82), Eosinophils (n = 82), Prothrombin time (n = 72), Activated partial thromboplastin time (n = 57), Ratio (n = 51), D-dimer (n = 46), INR (n = 72), Sodium (n = 81), Potassium (n = 78), Chlorine (n = 35), Calcium (n = 34), LDH (n = 64), AST (n = 39), ALT (n = 75), Total bilirubin (n = 42), C-reactive protein (n = 76), Ferritin (n = 65), Creatinine (n = 76), Troponin (n = 41), Urea (n = 47), Direct Bilirubin (n = 41), Gamma glutamyl transferase (n = 24), Fibrinogen (n = 32), Prothrombin (n = 34).

As shown in Table 3, comparison of laboratory parameters between the two groups demonstrates that patients with severe or critical disease have a significant decrease of lymphocytes (*p* = 0.001) and calcium (*p* = 0.006). The same patients have a significant increase of D-dimer (*p* = 0.021), LDH (*p*<0.0001), C-reactive protein (*p*<0.0001), ferritin (*p*<0.0001), urea (*p* = 0.013) and fibrinogen (*p* = 0.044).

Finally, multivariable logistic model confirmed age ≥ 50 years (50–65 vs 18–49; *p* = 0.005, OR = 3.23 CI95% 1.42–7.37) and high c-reactive protein (CPR) level (per unit of increase, *p*<0.0001, OR = 1.12 CI95% 1.06–1.20) as risk factors for severe or critical disease. Additionally, we found that patients in the severe or critical group showed a platelet count significantly lower (per 100 units of increase *p* = 0.010, OR = 0.61 CI95% 0.42–0.89) than those in the mild or moderate group (Table 4).

**Table 4:** The relationship between characteristics on admission of patients with SARS-CoV-2 infection and severe/critical disease: Multivariable logistic regression analysis.

Variable	OR (95%CI)
Age, < 50 vs ≥ 50 years	**3.23 (1.42–7.37)**
Platelets (per 100 units of increase)	**0.61 (0.42–0.89)**
C-reactive protein (per unit of increase)	**1.12 (1.06–1.20)**

**Fig 1:**
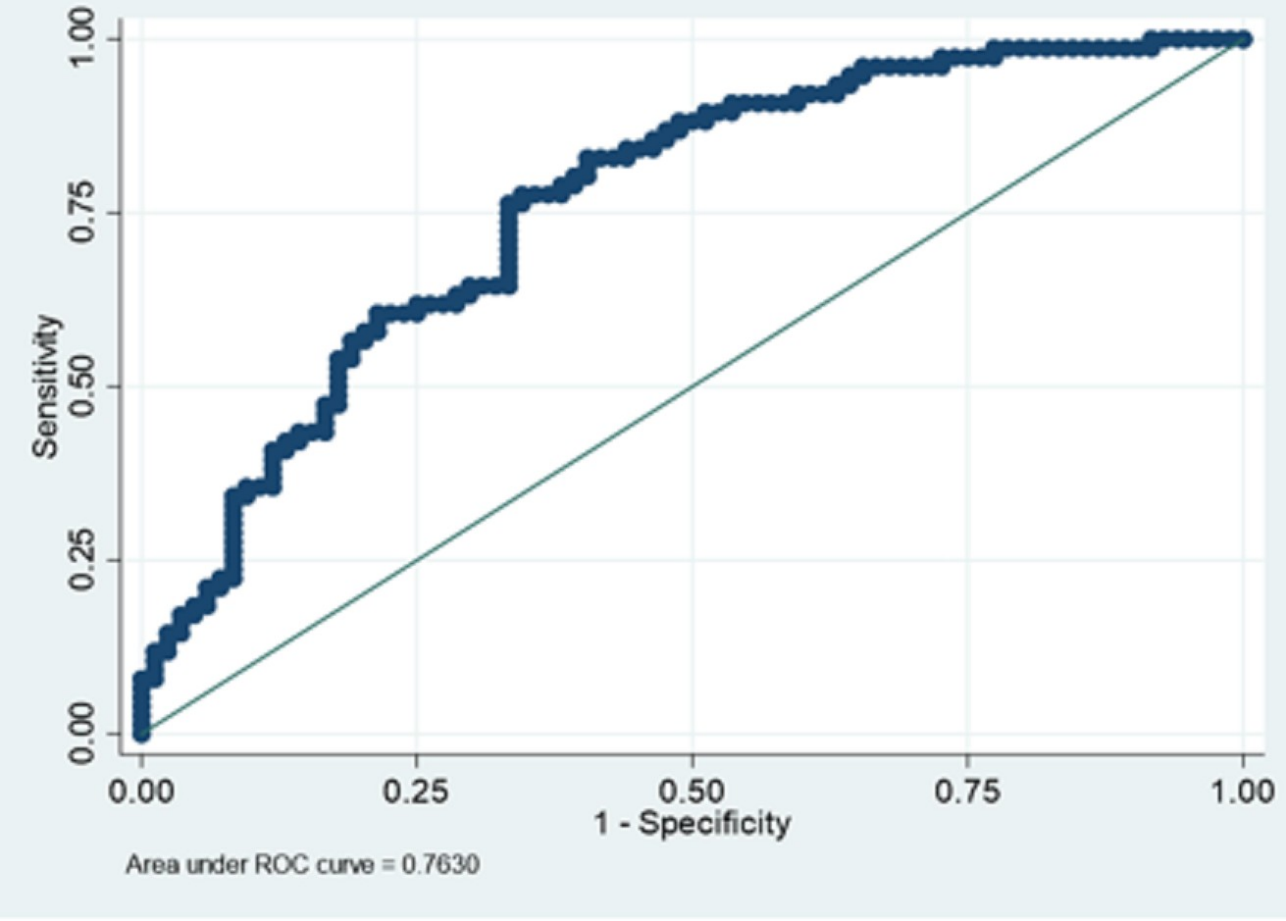
ROC curve of multivariable logistic regression model.

The multivariable logistic model showed a good discriminating capacity with a C-index value of 0.76 (Fig 1) and a bias-corrected C-index equal to 0.75. The calculated chi-square statistics for calibration is χ2 = 8.60; *p* = 0.38 which indicated goodness-of-fit of the model.

## Discussion

The present case series evaluated the clinical characteristics and prognostic factors of hospitalized patients aged ≤ 65 years affected by COVID-19. The difference between patients with mild or moderate disease and patients with severe or critical disease were analyzed. Patients aged ≤ 65 years with severe or critical illness represented 16% of all hospitalized COVID-19 patients. The findings of our study show that patients with severe or critical illness were more males than females and were older than patients with mild or moderate disease. Our results suggest that subjects in the age range which goes from 50 to 65 years old are more likely to develop a severe or critical COVID-19. Laboratory analysis at admission indicated that D-Dimer and C-reactive protein (CRP) levels were significantly higher in 82 patients with severe or critical disease than in 89 patients with mild or moderate disease. It is reasonable that these factors could predict COVID-19 severity in young patients. So far, it has been shown that CRP levels were correlated with severe SARS-CoV-2 infection in elderly patients [8, 11]. Moreover, severe COVID-19 has been shown to be associated with high D-dimer levels, which appear to predict mortality [16, 17]. High D-dimer and fibrinogen levels were also indicative of an increase in coagulation and consequently in venous thromboembolism [18–20]. In our case series fibrinogen was significantly higher in patients with severe or critical disease than patients with mild or moderate disease whereas no differences were found in prothrombin time but few data were available. Of 82 critically ill patients, three had a history of both pulmonary embolism and deep vein thrombosis and five showed only pulmonary embolism during hospitalization.

In our findings, a significant reduction of lymphocytes count was observed in patients with severe or critical disease at admission. A lymphocytopenia (count less than ≤ 1.0 x 10^9^/L as reported by Guan and colleagues) [21] was reported in 53% of all patients (45% group 1, 61% group 2). Lymphocytopenia is one of the most important hematological features in COVID-19 [21]. It is known that lymphocytopenia can be associated with progression disease [22] and increased mortality [7].

In laboratory findings, we also observed that an increment of per 100x10^3^/mcL in platelet count was significantly associated with a 40% decrease in severity disease. Several studies [7, 23, 24] have reported that low platelet count is associated with increased risk of severe disease and mortality in patients with COVID-19, and thus should serve to discriminate between severe and non-severe COVID-19 infections.

Other biomarkers of COVID-19 severity include lactate dehydrogenase (LDH) and serum levels of ferritin [25]. Elevated LDH levels in COVID-19 patients were associated with death [8, 18] and critical illness in elderly patients [7]. The present data show that LDH was significantly higher in patients with severe or critical disease considering the univariable logistic regression analysis. The meta-analysis of 10 studies demonstrated that ferritin level was associated with mortality and severe COVID-19 patients [26]. Our data showed a significantly increased of serum levels of ferritin in patients with severe or critical disease.

Albumin, urea and creatinine are clinical indicators of both kidney function and disease progression [27]. In our laboratory findings, only urea showed a significantly increased in patients with severe COVID-19.

Comparing electrolytes (sodium, potassium, chloride and calcium) between COVID-19 patients with and without severe disease only calcium was significantly lower in patients with severe COVID-19. A pooled analysis of five studies established COVID-19 severity is associated with lower serum concentrations of sodium, potassium and calcium [28].

Our study has several limitations. The sample size was small but most results were consistent with other studies on elderly patients with COVID-19. The study was carried out at a single center, thus the results may not be an exact representation of the general population, so we are planning to include the data of other centers for additional analyses. Since our case series derived from a retrospective study some relevant clinical data, such as IL6, fibrinogen, prothrombin, procalcitonin and creatin kinase were not available or available for few patients. Although several studies identified strong correlation between severe COVID-19 and cardiovascular and metabolic diseases [29, 30], our study didn’t find any statistically significant differences between these comorbidities and mild/moderate and severe/critical disease groups. Furthermore, this study didn’t evaluate the mortality rate in patients with severe COVID-19, thus a larger case series should be considered for further analysis.

To the best of our knowledge, this is the first study in which the clinical features were compared between two main clinical presentations in a young adult population (defined as 18 to 65 years old) of COVID-19 patients.

In conclusion, our findings highlight clinical features of patients infected with SARS-CoV2 and show that age ≥ 50 years, C-reactive protein levels and platelets count were independent prognostic factors of disease severity in COVID-19 patients aged ≤ 65 years. These predictor factors could help the clinicians to identify high-risk hospitalized patients in order to improve clinical management of the pandemic also among younger subjects.

## Supporting information

S1 Dataset (XLSX)

## References

[1] Hui DS, I Azhar E, Madani TA, Ntoumi F, Kock R, Dar O, et al. The continuing 2019-nCoV epidemic threat of novel coronaviruses to global health—The latest 2019 novel coronavirus outbreak in Wuhan, China. Int J Infect Dis. 2020;91: 264–266. 10.1016/j.ijid.2020.01.009 31953166 PMC7128332

[2] World Health Organization. Situation Report. 2020.

[3] Coronavirus disease (COVID-19)–World Health Organization. [cited 16 Nov 2020]. Available: https://www.who.int/emergencies/diseases/novel-coronavirus-2019

[4] COVID-19 ITALIA—Desktop. [cited 16 Nov 2020]. Available: http://opendatadpc.maps.arcgis.com/apps/opsdashboard/index.html#/b0c68bce2cce478eaac82fe38d4138b1

[5] Wu Z, McGoogan JM. Characteristics of and Important Lessons From the Coronavirus Disease 2019 (COVID-19) Outbreak in China: Summary of a Report of 72 314 Cases From the Chinese Center for Disease Control and Prevention. JAMA. 2020;323: 1239. 10.1001/jama.2020.2648 32091533

[6] Li J, Huang DQ, Zou B, Yang H, Hui WZ, Rui F, et al. Epidemiology of COVID-19: A systematic review and meta-analysis of clinical characteristics, risk factors, and outcomes. J Med Virol. 2020. 10.1002/jmv.26424 32790106 PMC7436673

[7] Wang L, He W, Yu X, Hu D, Bao M, Liu H, et al. Coronavirus disease 2019 in elderly patients: Characteristics and prognostic factors based on 4-week follow-up. J Infect. 2020. 10.1016/j.jinf.2020.03.019 32240670 PMC7118526

[8] Covino M, De Matteis G, Santoro M, Sabia L, Simeoni B, Candelli M, et al. Clinical characteristics and prognostic factors in COVID-19 patients aged ≥80 years. Geriatr Gerontol Int. 2020. 10.1111/ggi.13960 32516861 PMC7300699

[9] Stokes EK. Coronavirus Disease 2019 Case Surveillance—United States, January 22–May 30, 2020. MMWR Morb Mortal Wkly Rep. 2020;69. 10.15585/mmwr.mm6924e2 32555134 PMC7302472

[10] Zhang S-Y, Zhang Q, Casanova J-L, Su HC. Severe COVID-19 in the young and healthy: monogenic inborn errors of immunity? Nat Rev Immunol. 2020;20: 455–456. 10.1038/s41577-020-0373-7 32555547 PMC7302118

[11] Liu K, Chen Y, Lin R, Han K. Clinical features of COVID-19 in elderly patients: A comparison with young and middle-aged patients. J Infect. 2020;80: e14–e18. 10.1016/j.jinf.2020.03.005 32171866 PMC7102640

[12] World Health Organization. Laboratory testing for coronavirus disease 2019 (COVID-19) in suspected human cases. Interim guidance. 2020.

[13] Harvey LA. REDCap: web-based software for all types of data storage and collection. Spinal Cord. 2018;56: 625–625. 10.1038/s41393-018-0169-9 29977003

[14] Charlson ME, Pompei P, Ales KL, MacKenzie CR. A new method of classifying prognostic comorbidity in longitudinal studies: development and validation. J Chronic Dis. 1987;40: 373–383. 10.1016/0021-9681(87)90171-8 3558716

[15] World Health Organization. Clinical management of severe acute respiratory infection when novel coronavirus (2019-nCoV) infection is suspected Interim guidance 28 January 2020. 2020.

[16] Zheng Z, Peng F, Xu B, Zhao J, Liu H, Peng J, et al. Risk factors of critical & mortal COVID-19 cases: A systematic literature review and meta-analysis. J Infect. 2020. 10.1016/j.jinf.2020.04.021 32335169 PMC7177098

[17] Wool GD, Miller JL. The Impact of COVID-19 Disease on Platelets and Coagulation. Pathobiology. 2020; 1–13. 10.1159/000512007 33049751 PMC7649697

[18] Zhou F, Yu T, Du R, Fan G, Liu Y, Liu Z, et al. Clinical course and risk factors for mortality of adult inpatients with COVID-19 in Wuhan, China: a retrospective cohort study. The Lancet. 2020;395: 1054–1062. 10.1016/S0140-6736(20)30566-3 32171076 PMC7270627

[19] Artifoni M, Danic G, Gautier G, Gicquel P, Boutoille D, Raffi F, et al. Systematic assessment of venous thromboembolism in COVID-19 patients receiving thromboprophylaxis: incidence and role of D-dimer as predictive factors. J Thromb Thrombolysis. 2020; 1–6. 10.1007/s11239-020-02146-z 32451823 PMC7246965

[20] Ielapi N, Licastro N, Provenzano M, Andreucci M, Franciscis S de, Serra R. Cardiovascular disease as a biomarker for an increased risk of COVID-19 infection and related poor prognosis. Biomark Med. 2020;14: 713–716. 10.2217/bmm-2020-0201 32426991 PMC7236792

[21] Guan W, Ni Z, Hu Y, Liang W, Ou C, He J, et al. Clinical Characteristics of Coronavirus Disease 2019 in China. N Engl J Med. 2020; NEJMoa2002032. 10.1056/NEJMoa2002032 32109013 PMC7092819

[22] Ji D, Zhang D, Xu J, Chen Z, Yang T, Zhao P, et al. Prediction for Progression Risk in Patients With COVID-19 Pneumonia: The CALL Score. Clin Infect Dis. 2020;71: 1393–1399. 10.1093/cid/ciaa414 32271369 PMC7184473

[23] Lippi G, Plebani M, Henry BM. Thrombocytopenia is associated with severe coronavirus disease 2019 (COVID-19) infections: A meta-analysis. Clin Chim Acta. 2020;506: 145–148. 10.1016/j.cca.2020.03.022 32178975 PMC7102663

[24] Henry BM, de Oliveira MHS, Benoit S, Plebani M, Lippi G. Hematologic, biochemical and immune biomarker abnormalities associated with severe illness and mortality in coronavirus disease 2019 (COVID-19): a meta-analysis. Clin Chem Lab Med. 2020;58: 1021–1028. 10.1515/cclm-2020-0369 32286245

[25] Velavan TP, Meyer CG. Mild versus severe COVID-19: Laboratory markers. Int J Infect Dis. 2020;95: 304–307. 10.1016/j.ijid.2020.04.061 32344011 PMC7194601

[26] Huang I, Pranata R, Lim MA, Oehadian A, Alisjahbana B. C-reactive protein, procalcitonin, D-dimer, and ferritin in severe coronavirus disease-2019: a meta-analysis. Ther Adv Respir Dis. 2020;14: 1753466620937175. 10.1177/1753466620937175 32615866 PMC7336828

[27] Bao J, Li C, Zhang K, Kang H, Chen W, Gu B. Comparative analysis of laboratory indexes of severe and non-severe patients infected with COVID-19. Clin Chim Acta. 2020;509: 180–194. 10.1016/j.cca.2020.06.009 32511971 PMC7274996

[28] Lippi G, South AM, Henry BM. Electrolyte imbalances in patients with severe coronavirus disease 2019 (COVID-19). Ann Clin Biochem. 2020;57: 262–265. 10.1177/0004563220922255 32266828 PMC8173320

[29] Javanmardi F, Keshavarzi A, Akbari A, Emami A, Pirbonyeh N. Prevalence of underlying diseases in died cases of COVID-19: A systematic review and meta-analysis. PloS One. 2020;15: e0241265. 10.1371/journal.pone.0241265 33095835 PMC7584167

[30] Moazzami B, Chaichian S, Kasaeian A, Djalalinia S, Akhlaghdoust M, Eslami M, et al. Metabolic risk factors and risk of Covid-19: A systematic review and meta-analysis. PloS One. 2020;15: e0243600. 10.1371/journal.pone.0243600 33320875 PMC7737901

