# Supplementary material for: Baseline clinical characteristics and prognostic factors in hospitalized COVID-19 patients aged ≤ 65 years: A retrospective observational study
Source: PLoS One. 2021 Mar 24;16(3):e0248829. doi: 10.1371/journal.pone.0248829 (PMC7990225; doi:10.1371/journal.pone.0248829)
Supplement: Attachment — Submitted filename: Response to Reviewers.doc [file pone.0248829.s002.doc]

**Reply to Journal Requirements**

**Ethics statement**

We have added additional information both in the manuscript and in the online submission form about the patient records/samples used in the retrospective study.

In the Methods paragraph, Study design and participants section (page 4, lines 78-79) we have added the following sentence: “The study was conducted in accordance with the Declaration of Helsinki and the Good Clinical Practices guidelines for observational studies.”.

In the Methods paragraph, Procedures section we have reported the following sentences: “All data was pseudonymed according to clinical study and data protection regulations” (page 5, lines 91-92), “Patient’s medical records were accessed until July 2020.” (page 5, lines 112).

**Financial disclosure statement**

We apologize for the mistake but the financial disclosure statement previously reported was wrong.

Thus, we have changed the financial disclosure statement with “The authors received no specific funding for this work.”

**Data Availability statement**

As required, we have uploaded the study’s minimal underlying data set as Supporting Information file (S1_Data set) and we have added Supporting Information section at page 18 in the manuscript.

**Reply to Reviewer #1:**

**Comment**

The authors aimed to characterize Individual differences in susceptibility to SARS-CoV-2 infection, symptomatology and related clinical manifestations.

The paper is overall well written but in the Discussion section I would deepen a little bit the issues on cardiovascular disease and metabolic disease and covid-19 citing and discussing recent articles on these topics such as:

Ielapi N, et al. Cardiovascular disease as a biomarker for an increased risk of COVID-19 infection and related poor prognosis. Biomark Med. 2020;14(9):713-716.

Javanmardi F, et al. Prevalence of underlying diseases in died cases of COVID-19: A systematic review and meta-analysis. PLoS One. 2020 Oct 23;15(10):e0241265.

Moazzami B, et al. Metabolic risk factors and risk of Covid-19: A systematic review and meta-analysis. PLoS One. 2020 Dec 15;15(12):e0243600. doi: 10.1371/journal.pone.0243600. PMID: 33320875.

**Reply**

According to the reviewer's suggestion, we implemented the discussion on cardiovascular and metabolic diseases and COVID-19 by citing and discussing the suggested articles.

We have cited the article by Ielapi et al at page 14, line 14.

In the Discussion paragraph (page 15, lines 243-247) we have added the following sentence: “Although several studies identified strong correlation between severe COVID-19 and cardiovascular and metabolic diseases, our study didn’t find any statistically significant differences between these comorbidities and mild/moderate and severe/critical disease groups. Furthermore, this study didn’t evaluate the mortality rate in patients with severe COVID-19, thus a larger case series should be considered for further analysis.”
